# Prognostic Nomogram for Gastrointestinal Stromal Tumors after Surgery Based on the SEER Database

**DOI:** 10.1155/2022/5639174

**Published:** 2022-11-14

**Authors:** Qianhui Sun, Yunru Chen, Tingting Li, Xiaoyu Zhu, Guanghui Zhu, Baoyi Ni, Ruike Gao, Jie Li

**Affiliations:** ^1^Oncology Department, Guang'anmen Hospital, China Academy of Chinese Medical Sciences, Beijing, China 100053; ^2^Centre for Evidence-Based Chinese Medicine, Beijing University of Chinese Medicine, Beijing, China 100029; ^3^Beijing University of Chinese Medicine, Beijing, China 100029

## Abstract

We aimed to determine prognostic factors and develop an effective and practical nomogram for predicting cancer-specific survival in gastrointestinal stromal tumor (GIST) patients. Postoperative data were obtained from the SEER database (2000-2018). Patients were divided into training and validation cohorts at random (7 : 3). Prognostic factors were screened, and a prognostic nomogram was established using log-rank testing and Cox regression. We used DCA, ROC curves, C-index, and calibration curves to evaluate our model's predictive performance. The clinical value of the nomogram and the modified National Institute of Health (M-NIH) classification were compared using the NRI and IDI. The Kaplan-Meier method was applied to examine survival by risk group, and log-rank tests were applied to compare variations in survival curves. Independent prognostic risk factors associated with cancer-specific survival on multivariate Cox proportional hazards regression analysis were age, race, and tumor location, size, grade, and stage. Clinically relevant variables need to be considered in addition to statistically significant variables when developing prognostic models to aid clinical decision-making. We included two additional variables (mitotic rate and chemotherapy) when constructing the prognostic model. The C-index was 0.766 (95% confidence interval (CI): 0.737-0.794) in the training cohort and 0.795 (95% CI: 0.754-0.836) in the internal validation group suggesting robustness. The areas under the ROC curve for three-year and five-year survival were >0.700, indicating satisfactory discrimination. The calibration curves showed good agreement between the predictions of the nomogram and the actual results. The NRI (0.346 for 3-year and 0.265 for 5-year cancer-specific survival for patients with GIST (GSS) prediction; validation cohort: 0.356 for 3-year and 0.246 for 5-year GSS prediction) and IDI values (0.047 for 3-year and 0.060 for 5-year GSS prediction; validation cohort: 0.071 for 3-year and 0.084 for 5-year GSS prediction) suggested that the established nomogram performed significantly better than the M-NIH classification. The DCA indicated that the nomogram was clinically useful and had a high discriminative ability in identifying patients who were at high risk of poor outcomes. According to nomogram findings, patients were divided into three groups (high, moderate, and low risk), with significantly different prognoses in both cohorts. Our nomogram satisfactorily predicted survival in postsurgical GIST patients, which may assist clinicians to evaluate the postoperative status and guide subsequent treatments.

## 1. Introduction

The majority of gastrointestinal (GI) tract mesenchymal tumors, or GISTs, make about 18% of all sarcomas [1–3]. These tumors are derived from cells that are morphologically and immunophenotypically like Cajal cells [4]. GISTs are more prevalent in the stomach and small intestine, accounting for 60% and 30% of all sites, respectively [5, 6]. According to current epidemiologic evidence, the annual incidence of GISTs in the United States is estimated at 0.70 per 100,000 people and this incidence is rapidly increasing [7]. Novel treatment modalities have been developed with progress in molecular biology research on GISTs, although the standard treatment for GISTs is still surgery resection [8, 9]. Although most patients with GISTs have good postoperative prognoses, a high recurrence rate and gastrointestinal dysfunction remain unsettling problems [10–12]. Therefore, prognoses must be effectively determined in patients with GISTs after surgery.

Well-recognized prognostic factors include mitotic rate, tumor size, and primary tumor site, according to the most recent clinical guidelines, which include those from the National Comprehensive Cancer Network (NCCN), European Society for Medical Oncology (ESMO), European Reference Network on Rare Adult Cancers (EURACAN), and French Intergroup Clinical Practice guidelines [13–15]. These indicators continue to be the most frequently used prognostic variables in clinical practice and have been employed in the Armed Forces Institute of Pathology (AFIP), modified NIH (M-NIH), and National Institutes of Health (NIH) risk classification schemes. Other prognostic factors, such as depth of invasion, were sporadically related with long-term postoperative survival in patients with GISTs in addition to these risk groups [16–19]. As a result, there is still debate surrounding postoperative risk assessment for patients with GISTs.

Nomograms can assist in quantifying pathological and clinical features, as well as in the integration of these variables into a model estimating specific oncologic endpoints [20, 21]. Predictive nomograms may help patients and physicians make optimal treatment decisions by establishing a direct assessment system. Moreover, the NCCN has described the use of nomograms to evaluate prognoses in patients with GISTs. However, nomograms for predicting prognoses and guiding postoperative treatment of GISTs are rare.

The demographic and incidence data collected by the Surveillance, Epidemiology, and End Results (SEER) registry cover approximately 28% of the U.S. population and are considered representative of the entire U.S. population. We aimed to use known clinicopathological characteristics obtained from the SEER database to create a nomogram for predicting postoperative survival risk in patients with GISTs. We assessed the nomogram's precision and validated it with a validation set.

## 2. Materials and Methods

### 2.1. Data Source and Patient Selection

Data were retrieved from the 18 SEER program population-based registries' November 2020 submittal. As previously mentioned, SEER gathers data from 18 U.S. registries on cancer incidence, mortality, population-based variables, main tumor features, and clinical management [22].

**Figure 1:**
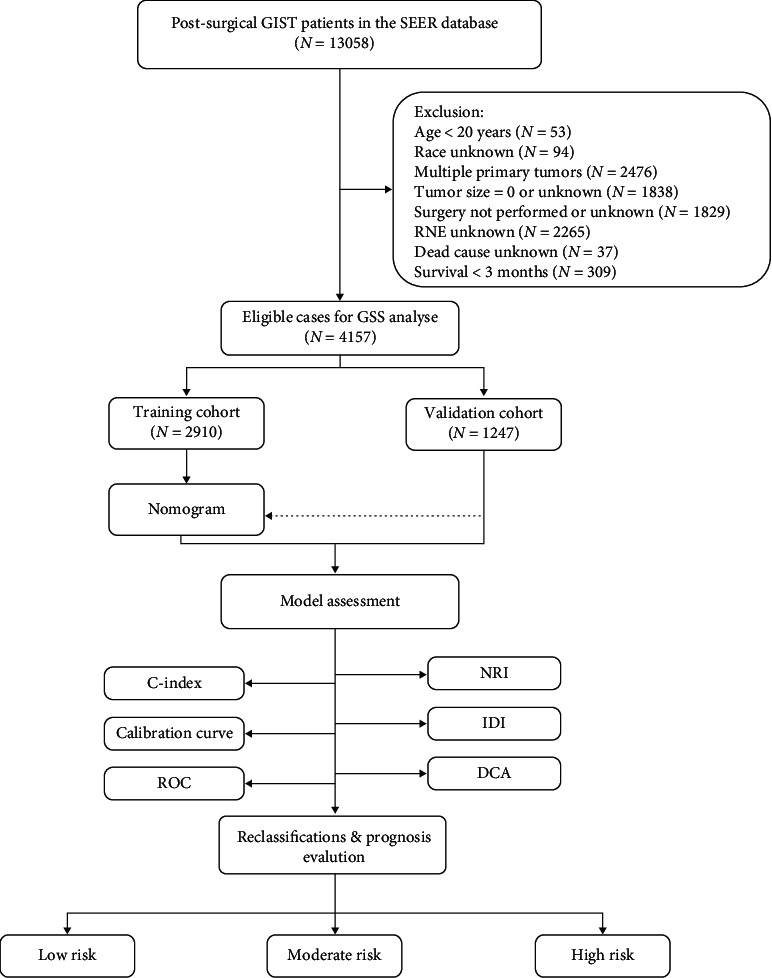
Workflow chart. DCA: decision curve analysis; GIST: gastrointestinal stromal tumor; IDI: integrated discrimination improvement; NRI: net reclassification index; RNE: regional nodes examined; ROC curve: receiver operating characteristic curve.

We used the SEER Stat software (version 8.3.9.2) to extract data on patients diagnosed with GIST between 2000 and 2018.  Figure 1 illustrates the patient selection procedure. Only patients with primary GISTs were included. Patients who met the criteria below were excluded: (1) age < 20 years at diagnosis; (2) not having undergone resection at the primary site, or missing surgical records; (3) multiple primary tumors; (4) missing data on race, tumor size, regional nodes examined (RNE), or cause of death; and (5) survival of <3 months.

This study conformed with the principles of the Declaration of Helsinki and was considered exempt by the Institutional Review Board at Guang'anmen Hospital (under China Academy of Chinese Medical Sciences) because the SEER data contained deidentified information.

### 2.2. Study Design and Statistical Analyses

Patients with GIST who met the study inclusion criteria were randomly assigned to a training group (*n* = 2,910) or a validation group (*n* = 1,247) in a 7 : 3 ratio using the R function “createDataPartition.” A training cohort is used to filter variables and build a model, whereas a validation cohort is used to double-check the training cohort's results. Eleven SEER database variables were used: age at diagnosis, sex, race, tumor location, size, grade, American Joint Committee on Cancer (AJCC) stage, mitotic rate, surgery, RNE, and chemotherapy. Surgery types included local and radical excision. Tumor stage was recorded based on the AJCC eighth edition prognostic staging guidelines, and this staging system provides more reliable prognostic information than those provided by anatomic staging.

The study's primary endpoint was cancer-specific survival in patients with GISTs (GSS: cancer-specific survival for patients with GISTs). More specifically, GSS was defined as the period between GIST diagnosis and cancer-related death as reported by the registry. Other deaths not related to GIST were censored. The censoring was estimated based on the SEER database's coding of these endpoints (alive, cancer-related death, or death from other causes).

R statistical software was utilized for all analyses (version 4.1.0, Vienna, Austria). A two-sided *P* value of 0.05 was used to define statistical significance. The median (interquartile range (IQR)) and *n* (percent) were used to represent continuous and categorical data, respectively. Moreover, GSS was computed using the Kaplan-Meier estimator following a descriptive analysis, and log-rank testing was used to compare the Kaplan-Meier curves. Univariate and multivariate Cox regression analyses were conducted on all 11 variables, with variables showing a *P* value of < 0.05 on multivariate Cox regression recognized as independent risk factors.

A final nomogram was developed based on clinical and statistical importance and was confirmed both internally and externally using a bootstrapping technique [23]. The C-index and the integrated area under the curve (AUC) generated with bootstrapping were utilized to compare this nomogram to the M-NIH classification. Calibration plots were established to evaluate model calibration. Currently, the net reclassification index (NRI), integrated discrimination improvement (IDI), and decision curve analysis (DCA) are used to evaluate the clinical utility of alternative models [24, 25]. By calculating net benefits at different threshold probabilities, these techniques are applied to nomograms. Using a bootstrap sample of 1,000 replicates taken from the original data, the NRI, IDI, and their associated 95% confidence intervals (CIs) [26] were all computed. The Kaplan-Meier method was used to compare risk stratifications based on the nomogram and the M-NIH criteria. Utilizing X-tile, the cutoff point for risk stratifications was established [27].

## 3. Results

### 3.1. Characteristics of the Training and Validation Groups

1,247 patients made up the validation set, whereas 2,910 patients made up the training set.  Table 1 presents a summary of the included patients' initial characteristics. The median survival time of the training cohort was 45.0 (22.0–73.0) months. The stomach was the main site of GIST occurrence. Patients at AJCC stage I with no RNEs accounted for 44.4% and 70.4% of cases in the training and validation sets, respectively. Moreover, most patients had undergone radical excision (*n* = 2,515, 86.4%) without having undergone chemotherapy or had an unknown chemotherapy status (*n* = 1,633, 56.1%).

### 3.2. Screening for Independent Informative Prognostic Variables

**Figure 2:**
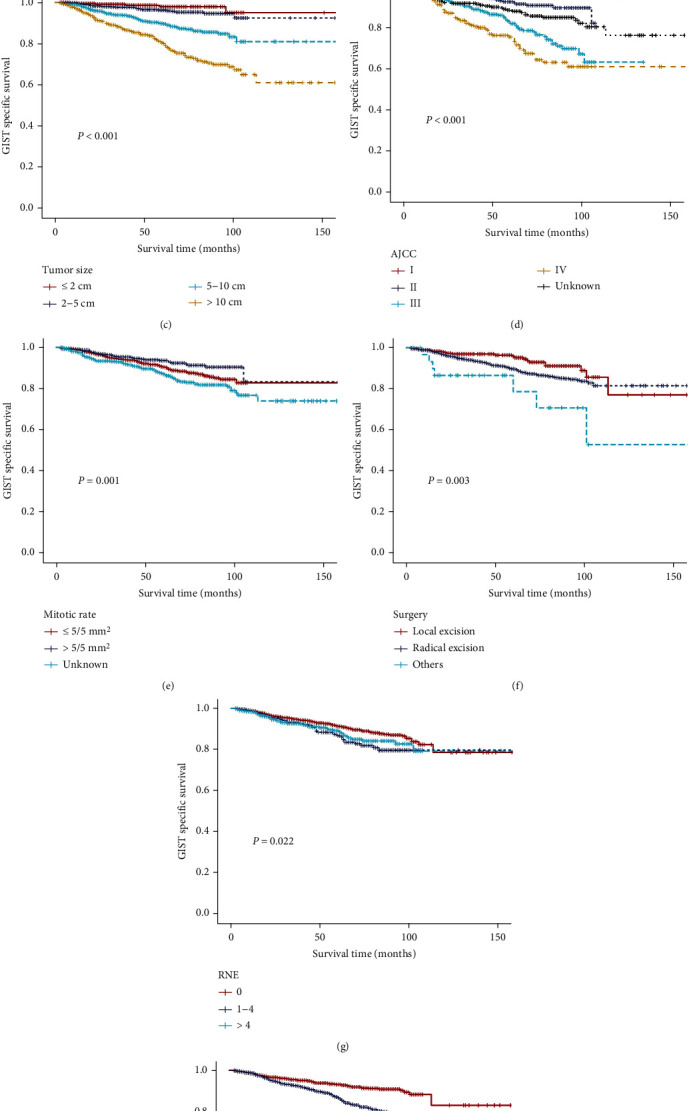
Kaplan-Meier GSS curves stratified by patient characteristics in the training set: (a) tumor location, (b) tumor grade, (c) tumor size, (d) AJCC stage, (e) mitotic rate, (f) surgery, (g) RNE, and (h) chemotherapy. GSS: cancer-specific survival for patients with GISTs; RNE: regional nodes examined.

Univariate and multivariate analyses were conducted to screen predictors of GSS among the 2,910 patients in the training cohort. All statistically significant values are shown in Figure 2. Tumor size and location, surgery, RNE, tumor grade, mitotic rate, AJCC stage, and chemotherapy were correlated with GSS in univariate survival analyses using the Kaplan-Meier method. These factors were thoroughly compared using log-rank tests. Moreover, a Cox proportional hazards regression model was utilized to precisely assess the impacts of different factors. The preliminary prognostic factors (variables showing a *P* value < 0.1 in the univariate Cox analyses) were included in the multivariate Cox regression model. Finally, six variables (age, race, tumor size, location, grade, and AJCC stage) were independent prognostic factors of GSS for patients with GISTs following surgery (Table 2).

**Figure 3:**
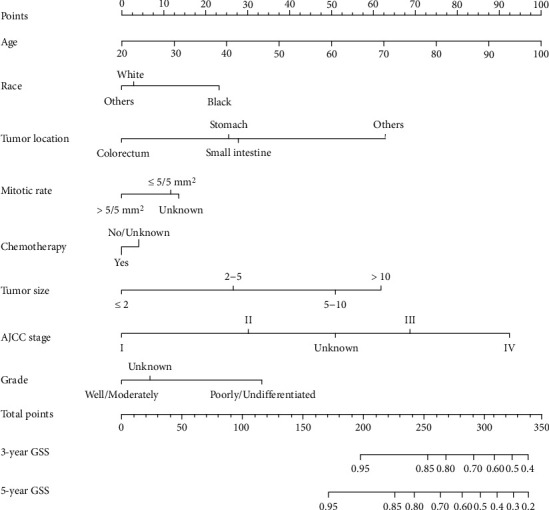
Nomogram for predicting three-year and five-year GSS in patients with GISTs.

Developing prognostic models to aid in clinical decision-making may require accounting for not only statistically significant variables but also for clinically relevant, statistically nonsignificant variables. Therefore, eight variables were applied to create a nomogram of the three-year and five-year GSS in the current study (Figure 3). The top four factors for the nomogram model were age, AJCC stage, tumor location, and tumor size. The anticipated three-year and five-year GSS probabilities were easily determined by adding the scores associated with each variable and projecting the sums to the bottom scales.

### 3.3. Validation of the Prognostic Nomogram

**Figure 4:**
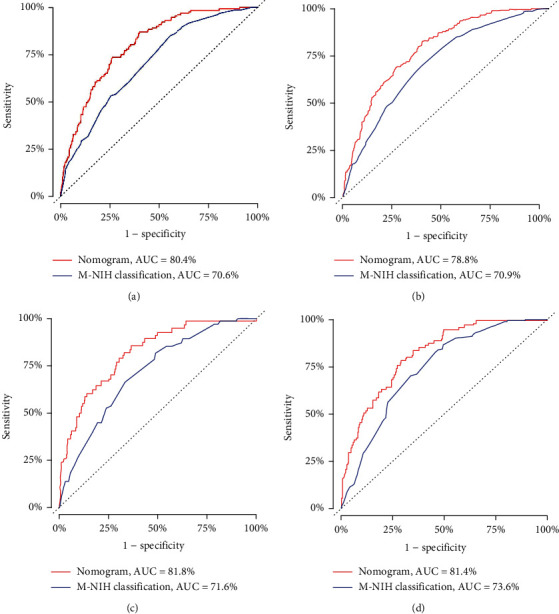
AUC values of ROCs for the nomogram and M-NIH classifications. (a, b) Training group: three-year GSS (80.4% vs. 70.6%) and five-year GSS (78.8% vs. 70.9%). (c, d) Validation group: three-year GSS (81.8% vs. 71.6%) and five-year GSS (81.4% vs. 73.6%). AUC: area under the ROC; GISTs: gastrointestinal stromal tumors; M-NIH classification: modified NIH classification; ROC curve: receiver operating characteristic curve.

**Figure 5:**
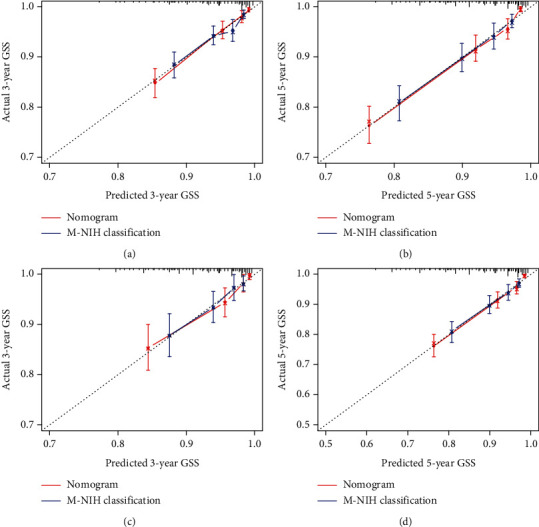
Calibration curves for the nomogram (red lines) and M-NIH (blue lines) classifications. (a, b) Training group: predicting three-year and five-year GSS. (c, d) Validation group: predicting three-year and five-year GSS.

In the training set, the C-indices of the GSS nomogram were 0.766 (95% CI, 0.737-0.794), while in the validation set, they were 0.795 (95% CI, 0.754-0.836) (Table 3). The AUCs at three and five years were 0.804 and 0.788 in the training group and 0.818 and 0.814 in the validation group, respectively, according to the time-dependent receiver operating characteristic curves for GSS (Figure 4). The calibration curves showed excellent agreement between the predictions of the nomogram and actual observations in both the training and validation sets for the three-year and five-year GSS survival probabilities (Figure 5).The GIST nomogram exhibited considerable discriminative and calibration power.

**Figure 6:**
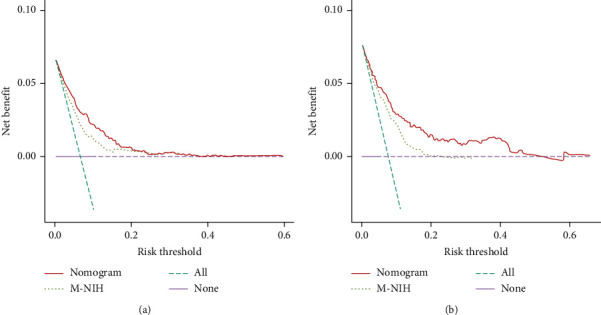
Decision curve analysis (DCA) for the nomogram and M-NIH classification. (a) DCA of the training group. (b) DCA of the validation group. AUC: area under the receiver operating characteristic curve.

The accuracy of the nomogram and M-NIH categorization were compared according to the changes in the C-index as well as the NRI and IDI values. While applying the nomogram in the training cohort (Table 3), the C-index was 0.051 (95% CI = 0.020–0.073, *P* < 0.001), the NRI values for the three- and five-year GSS were 0.346 (95% CI = 0.198–0.472) and 0.265 (95% CI = 0.157–0.405), and the IDI values for the three-year and five-year GSS were 0.047 (95% CI = 0.031–0.083, *P* < 0.001) and 0.060 (95% CI = 0.041–0.099, *P* < 0.001). These results were confirmed in the validation cohort (Table 3), indicating that the nomogram predicted prognoses with greater accuracy than the M-NIH classification. Furthermore, DCA curves are plotted in Figure 6; the nomogram showed net benefits in threshold probabilities over a wider range than that associated with the M-NIH classification in both the training and validation sets.

### 3.4. Risk Stratification

**Figure 7:**
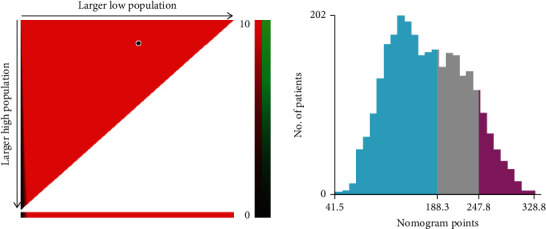
Cutoff values were calculated using X-tile based on total scores.

**Figure 8:**
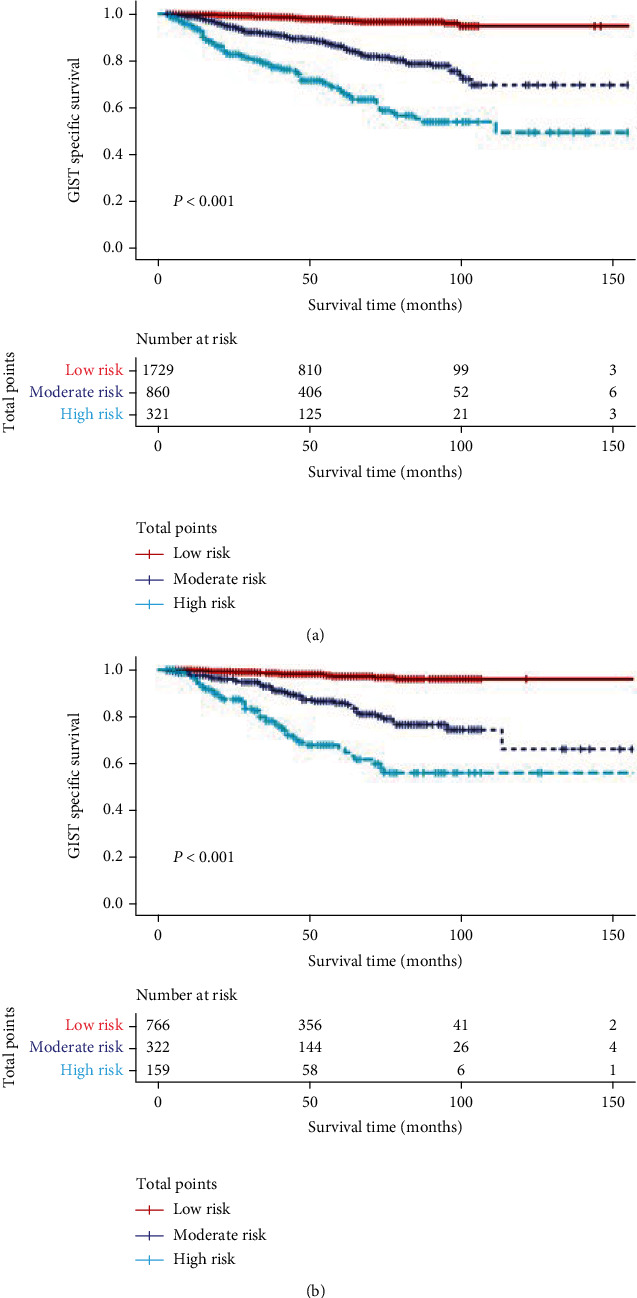
Survival curves for different risk groups according to total scores (188.3 and 247.8). (a) Survival curves for the training group. (b) Survival curves for the validation group.

The optimum cutoff value was calculated using X-tile based on the overall scores for our defined nomogram. Patients with GISTs were categorized as low risk (score ≤ 188.3), moderate risk (188.3 < score ≤ 247.8), or high risk (score > 247.8) (Figure 7). After that, the Kaplan-Meier survival curves were produced, as shown in Figure 8. In both cohorts, the prognoses for the high-risk, moderate-risk, and low-risk groups were statistically substantially different (log-rank test, *P* < 0.001).

## 4. Discussion

There is a substantial chance of recurrence after surgical resection, despite the fact that it is still the primary treatment for primary GISTs. Furthermore, there is limited information about the postoperative prognosis of GISTs because of their rarity. To our knowledge, this study assessed the biggest group of postsurgical GIST patients. Using the SEER database, we discussed this tumor's prognostic characteristics. Our study established and effectively validated a prognostic GSS nomogram for patients with GISTs following surgery, which can be integrated into clinical practice to guide treatment decisions and prognostic evaluations based on tumor and demographic characteristics. Six variables selected using Cox regression analysis were incorporated into the nomogram along with several clinicopathological features. Age was the most important prognostic factor, as measured using standard deviations along with nomogram scales, followed by AJCC stage, tumor location, and tumor size.

The site distribution of GISTs in the current study closely matched the findings of the previous studies; the stomach was the most common, followed by the small intestine and other sites [16, 28]. Unlike earlier findings [29, 30], a colorectal tumor location was the best prognostic factor among the evaluated GIST sites in this study. Previous studies have confirmed that the risk of colorectal GISTs is comparable to that of small intestine GISTs [18, 31] and is higher than that of stomach GISTs. However, owing to the small sample size, the current analysis of colorectal GISTs might have been underpowered [16, 31] and this question requires further investigation. Similarly, the ranking (in descending order) of the three other tumor sites was as follows: “other,” small intestine, and stomach.

Gastric GISTs (G-GISTs) are usually small in size, and the pathogenesis of *KIT* exon 9 mutations mainly occurs at nongastric sites; this may lead to worse clinical outcomes and necessitate higher doses of imatinib as adjuvant therapy [32]. Regarding the small intestine site, a multicenter study found that mutations in exons of *KIT* 13 and 17 (which occur predominantly in the small intestine) are often associated with larger, more aggressive GISTs [16, 33]. GISTs at other sites are extragastrointestinal, and most of these tumors likely have poor prognoses because they represent metastases from the gastrointestinal tract [29, 34].

Moreover, the AJCC tumor stages were statistically significantly associated with postoperative GSS. AJCC stage alone is a clinically established benchmark for survival prediction. AJCC 8th edition classification adds tumor quantity, tumor size, lymph node metastasis, and vascular invasion as new staging parameters. This may represent one reason this marker is associated with GIST-related mortality risk. However, AJCC staging is infrequently used as a biomarker in clinical practice.

Although the M-NIH criteria, which primarily focus on the risk of recurrence, are among the most commonly accepted risk categorization systems, our developed nomogram revealed clear advantages over the M-NIH criteria in the current investigation. The nomogram showed higher sensitivity, specificity, and believability than the used M-NIH criteria, according to the C-index, time-dependent AUC, and calibration curves computed by bootstrapping, which were adequate in both the training and validation populations. Moreover, the DCA curves demonstrated that the nomogram facilitated better clinical decision-making. As an important component of the M-NIH criteria, mitotic rate was unexpectedly identified as an independent prognostic factor when analyzed along with tumor size and location in the multivariate analysis, demonstrating that the mitotic rate may not statistically significantly affect cancer-specific survival; however, the mitotic rate was associated with GIST recurrence risk. The nomogram also considered the weighting of the mitotic rate, tumor size, and tumor location. We suggest that treatment strategies and demographic information should be taken into account via a prognostic score system and that this would likely improve predictive performance and optimize clinical decision-making.

Surgical resection is the first choice for the treatment of GISTs, with approximately 45% to 60% of GISTs undergoing R0 resection [35, 36]. This study's results may help reconcile previous controversial findings on the role of the surgery type in the postoperative prognosis of GISTs. More specifically, the present study did not support the use of this parameter as an independent risk factor in clinical risk staging or its use as an independent risk factor in a clinical risk assessment system. Other reports suggested that R1 and R0 resections show similar prognoses [35, 37], whereas another study reported contrasting findings. More specifically, a retrospective study including 129 patients revealed higher recurrence-free and disease-specific survival in patients undergoing R0 surgery compared with those undergoing R1 resection, but no statistically significant difference between the two groups was found following wide surgical margin exclusion [38]. Additionally, gastrointestinal bleeding [39] and tumor rupture [40] during surgery purportedly contribute to a poor prognosis for patients with GISTs. Notably, like the previous reports [41, 42], age was the most important risk factor for GSS, indicating that surgery may not be a good choice for elderly adults with GISTs.

Despite the generated nomogram performing well, there were a number of limitations in the current investigation, which we mention here. For example, the SEER database lacked details on surgical margins, tumor rupture and bleeding, mutation types, adjuvant therapy, and the administration of tyrosine kinase inhibitors. Therefore, we did not investigate these variables. Moreover, a large-scale prospective multicenter clinical validation investigation could help to address the inherent and unavoidable biases that this study's retrospective methodology was prone to.

## 5. Conclusions

Given its increased accuracy, good clinical utility, and more precise prognosis prediction compared with those of conventional risk classifications, our nomogram may be used to predict cancer-specific survival in patients with GISTs following surgery. More specifically, this can aid clinicians to assess the postoperative situation of patients with GISTs and to implement more effective treatment.

## 6. Database Selection Description

At the beginning of this paper, we used SEER Research Plus Data, 17 Registries, Nov 2021 Sub (2000-2019), which was officially released on the official website in April 2022 and checked in November 2021. However, through the inclusion and exclusion screening of SEER Research Plus Data, 17 Registries, Nov 2021 Sub (2000-2019), it was finally found that the diagnosis year of the included case Data was in the period of 2000-2018. We then tried to use another database from 2000 to 2018: SEER Research Plus Data, 18 Registries, Nov 2020 Sub. This database was derived from 18 registries, which is more than the 17 registries in the last database. A wide range of case information collection sources may lead to more accurate and realistic research results. Therefore, SEER Research Plus Data, 18 Registries, Nov 2020 Sub was selected for this study.

## Figures and Tables

**Table 1:** Characteristics of patients with gastrointestinal stromal tumors in the training and validation groups.

Characteristic	Whole population	Training cohort	Validation cohort
Total patients	4157	2910	1247
Age at diagnosis (years)	63 (53, 71)	63 (53, 71)	63 (53, 72)
Sex			
Male	2017 (48.5)	1420 (48.8)	597 (47.9)
Female	2140 (51.5)	1490 (51.2)	650 (52.1)
Race			
White	2799 (67.3)	1950 (67.0)	849 (68.1)
Black	740 (17.8)	521 (17.9)	219 (17.6)
Other	618 (14.9)	439 (15.1)	179 (14.4)
Tumor location			
Stomach	2625 (63.1)	1840 (63.2)	785 (63.0)
Small intestine	1207 (29.0)	845 (29.0)	362 (29.0)
Colorectum	132 (3.2)	93 (3.2)	39 (3.1)
Other	193 (4.6)	132 (4.5)	61 (4.9)
Tumor grade			
Well/moderately differentiated	1640 (39.5)	1143 (39.3)	497 (39.9)
Poorly differentiated/undifferentiated	460 (11.1)	329 (11.3)	131 (10.5)
Unknown	2057 (49.5)	1438 (49.4)	619 (49.6)
Tumor size			
≤2 cm	526 (12.7)	372 (12.8)	154 (12.3)
2-5 cm	1360 (32.7)	939 (32.3)	421 (33.8)
5-10 cm	1300 (31.3)	908 (31.2)	392 (31.4)
>10 cm	971 (23.4)	691 (23.7)	280 (22.5)
AJCC stage			
I	1867 (44.9)	1291 (44.4)	576 (46.2)
II	661 (15.9)	470 (16.2)	191 (15.3)
III	701 (16.9)	510 (17.5)	191 (15.3)
IV	450 (10.8)	299 (10.3)	151 (12.1)
Unknown	478 (11.5)	340 (11.7)	138 (11.1)
Mitotic rate			
≤5/5 mm^2^ HPF	2256 (54.3)	1573 (54.1)	683 (54.8)
>5/5 mm^2^ HPF	932 (22.4)	648 (22.3)	284 (22.8)
Unknown	969 (23.3)	689 (23.7)	280 (22.5)
Surgery			
Local excision	516 (12.4)	363 (12.5)	153 (12.3)
Radical excision	3594 (86.5)	2515 (86.4)	1079 (86.5)
Other	47 (1.1)	32 (1.1)	15 (1.2)
RNE			
0	2911 (70.0)	2050 (70.4)	861 (69.0)
1-4	599 (14.4)	414 (14.2)	185 (14.8)
>4	647 (15.6)	446 (15.3)	201 (16.1)
Chemotherapy			
No/unknown	2323 (55.9)	1633 (56.1)	690 (55.3)
Yes	1834 (44.1)	1277 (43.9)	557 (44.7)
Survival (months)	45 (22, 73)	45 (22, 73)	45 (21, 72)

AJCC stage: American Joint Committee on Cancer stage; HPF: high-power microscopic fields; RNE: regional nodes examined.

**Table 2:** Univariate and multivariate Cox regression analyses of GSS from the constructed nomogram.

Variable	Univariate analysis			Multivariate analysis		
	HR	95% CI	*P* value	HR	95% CI	*P* value
Age at diagnosis (years)	1.020	1.010-1.030	<0.001	1.026	1.015-1.037	<0.001
Sex						
Male						
Female	0.824	0.640-1.060	0.132			
Race						
White						
Black	1.350	0.993-1.850	0.055	1.518	1.096-2.102	0.012
Other	0.974	0.666-1.420	0.892	0.955	0.651-1.401	0.812
Tumor location						
Stomach						
Small intestine	1.630	1.230-2.160	<0.001	1.046	0.770-1.422	0.773
Colorectum	0.813	0.332-1.990	0.651	0.626	0.241-1.626	0.336
Other	3.900	2.700-5.630	<0.001	2.174	1.408-3.358	<0.001
Tumor grade						
Well/moderately differentiated						
Poorly differentiated/undifferentiated	4.570	3.230-6.460	<0.001	1.995	1.383-2.877	<0.001
Unknown	1.550	1.120-2.140	<0.001	1.143	0.815-1.603	0.437
Tumor size						
≤2 cm						
2-5 cm	1.930	0.803-4.660	0.142	1.662	0.683-4.045	0.263
5-10 cm	5.450	2.370-12.500	<0.001	2.732	1.142-6.537	0.024
>10 cm	11.400	5.020-25.800	<0.001	3.351	1.382-8.124	0.007
AJCC stage						
I						
II	2.950	1.720-5.060	<0.001	1.883	1.043-3.400	0.036
III	8.320	5.310-13.100	<0.001	4.088	2.339-7.142	<0.001
IV	12.800	8.090-20.200	<0.001	6.771	3.832-11.961	<0.001
Unknown	5.030	3.050-8.320	<0.001	2.849	1.547-5.248	0.001
Mitotic rate						
≤5/5 mm^2^ HPF						
>5/5 mm^2^ HPF	0.690	0.475-1.000	0.051	0.792	0.542-1.156	0.227
Unknown	1.400	1.060-1.850	0.018	1.006	0.696-1.453	0.976
Surgery						
Local excision						
Radical excision	1.680	1.070-2.640	0.023	1.103	0.681-1.784	0.691
Other	4.070	1.730-9.570	0.001	1.967	0.818-4.726	0.130
RNE						
0						
1-4	1.520	1.090-2.110	0.014	1.213	0.861-1.710	0.270
>4	1.350	0.969-1.890	0.076	0.928	0.658-1.310	0.671
Chemotherapy						
No/unknown						
Yes	2.000	1.540-2.590	<0.001	0.919	0.688-1.227	0.568

AJCC stage: American Joint Committee on Cancer stage; CI: confidence interval; gastrointestinal stromal tumors; GSS: cancer-specific survival for patients with GISTs; HR: hazard ratio.

**Table 3:** Performance of the derived nomogram and M-NIH classification in patients with GISTs.

Index	Training cohort			Validation cohort		
	Estimate	95% CI	*P* value	Estimate	95% CI	*P* value
NRI (vs. M-NIH classification)						
3-year GSS	0.346	0.198-0.472		0.356	0.171-0.551	
5-year GSS	0.265	0.157-0.405		0.246	0.087-0.491	
IDI (vs. M-NIH classification)						
3-year GSS	0.047	0.031-0.083	<0.001	0.071	0.032-0.122	<0.001
5-year GSS	0.060	0.041-0.099	<0.001	0.084	0.040-0.142	<0.001
C-index						
Nomogram	0.766	0.737-0.794		0.795	0.754-0.836	
M-NIH classification	0.714	0.681-0.747		0.732	0.687-0.778	
Change	0.051	0.020-0.073	<0.001	0.063	0.026-0.090	<0.001

C-index: concordance index; CI: confidence interval; GISTs: gastrointestinal stromal tumors; IDI: integrated discrimination improvement; M-NIH classification: modified NIH classification; NRI: net reclassification improvement.

## Data Availability

Population-based research was retrospectively performed with data from the SEER database, which incorporates national information on tumor samples from 18 large-scale cancer registries and is open to public for cancer studies (https://seer.cancer.gov/).
